# Supplementary material for: Unveiling fatal risk factors: Predicting hemophagocytic lymphohistiocytosis in SFTS patients
Source: PLoS Negl Trop Dis. 2025 Jun 24;19(6):e0013207. doi: 10.1371/journal.pntd.0013207 (PMC12186921; doi:10.1371/journal.pntd.0013207)
Supplement: S1 Table — (DOCX) [file pntd.0013207.s001.docx]

**Supplementary Table 1 Comparison of clinical characteristics and laboratory markers at admission for SFTS patients with HLH between the Surviving and Deceased Groups**

| **Parameters** | **Total(n=53)** | **Survival (n=28)** | **Deceased (n=25)** | ***P*** |
| --- | --- | --- | --- | --- |
| **Sex ,N(%)** |  |  |  | 0.506 |
| Female | 28(52.8) | 16(57.1) | 12(48.0) |  |
| Male | 25(47.2) | 12(42.9) | 13(52.0) |  |
| **Age ,Y,mean(±SD)** | 63.0±9.5 | 58.3±8.9 | 68.3±7.0 | **<0.001** |
| **Area ,N(%)** |  |  |  | 0.201 |
| Urban area | 5(9.4) | 4(14.3) | 1(4.0) |  |
| Rural area | 48 (90.6) | 24(85.7) | 24(96.0) |  |
| **Season of onset ,N(%)** |  |  |  | 0.859 |
| Spring and summer | 29(54.7) | 15(53.6) | 14(56.0) |  |
| Autumn and winter | 24(45.3) | 13(46.5) | 11(44.0) |  |
| **Symptoms,N(%)** |  |  |  |  |
| Fever | 50(94.3) | 28(100.0) | 22(88.0) | 0.059 |
| Muscular soreness | 9(17.0) | 5(17.9) | 4(16.0) | 0.857 |
| cough | 6(11.3) | 1(3.6) | 5(20.0) | 0.060 |
| Weakness | 21(39.6) | 12(42.9) | 9(36.0) | 0.610 |
| Inappetence | 19(35.9) | 10(35.7) | 9(36.0) | 0.983 |
| Nausea | 12(22.6) | 5(17.9) | 7(28.0) | 0.378 |
| Vomiting | 13(24.5) | 6(21.4) | 7(28.0) | 0.579 |
| Abdominal pain | 3(5.7) | 2(7.1) | 1(4.0) | 0.621 |
| Diarrhea | 14(26.4) | 7(25.0) | 7(28.0) | 0.805 |
| Headache and dizziness | 21(39.6) | 9(32.1) | 12(48.0) | 0.239 |
| Consciousness disorder | 3(5.7) | 1(3.6) | 2(8.0) | 0.486 |
| **Comorbidities ,N(%)** |  |  |  |  |
| hypertension | 18(34.0) | 7(25.0) | 11(44.0) | 0.145 |
| Diabetes mellitus | 19(35.9) | 9(32.1) | 10(40.0) | 0.552 |
| Cerebrovascular diseases | 9(17.0) | 4(14.3) | 5(20.0) | 0.580 |
| Lung disease | 3(5.7) | 0(0.0) | 3(12.0) | 0.059 |
| Chronic kidney disease | 1(1.9) | 0(0.0) | 1(4.0) | 0.285 |
| **Therapy, N(%)** |  |  |  |  |
| Corticosteroid | 45(84.9) | 22(78.6) | 23(92.0) | 0.173 |
| Intravenous immunoglobulin | 25(47.1) | 10(35.7) | 15(60.0) | 0.077 |
| continuous renal replacement therapy | 26(49.1) | 12(42.9) | 14(56.0) | 0.339 |
| Respiratory support | 11(20.8) | 1(3.6) | 10(40.0) | **0.001** |
| **History of tick bite, N(%)** | 13(24.6) | 9(32.1) | 4(16.0) | 0.173 |
| **Time from onset to admission ,d,median[IQR]** | 6.0[4.0,7.0] | 7.0[5.0,7.0] | 5.0[4.0,7.0] | 0.104 |
| **Duration of hospital admission ,d,median[IQR]** | 17.4±8.8 | 21.9±9.1 | 12.5±5.0 | **<0.001** |
| **Blood routine indicators** |  |  |  |  |
| WBC,×10^9^/L | 2.45[1.61,4.21] | 3.21[1.92,5.42] | 2.10[1.09,3.19] | 0.051 |
| Lymphocyte ,×10^9^/L | 0.45[0.32,0.85] | 0.52[0.33,0.92] | 0.45[0.30,0.59] | 0.382 |
| Monocyte,×10^9^/L | 0.09[0.06,0.13] | 0.10[0.09,0.25] | 0.08[0.05,0.11] | **0.032** |
| Neutrophil,×10^9^/L | 1.72[1.00,3.33] | 1.95[1.17,3.88] | 1.58[0.76,2.29] | 0.121 |
| Platelet,×10^9^/L | 33.0[25.0,52.0] | 33.0[27.0,52.0] | 34.0[21.0,46.0] | 0.428 |
| RBC,×10^12^/L | 4.22±0.67 | 4.31±0.60 | 4.13±0.72 | 0.337 |
| Hemoglobin,g/L | 128.23±21.09 | 130.57±18.20 | 125.60±23.64 | 0.401 |
| **Blood biochemistry indicators** |  |  |  |  |
| ALT, U/L | 116.0[70.0,193.0] | 150.0[62.0,206.0] | 108.0[75.0,146.0] | 0.219 |
| AST, U/L | 357.0[224.0,677.0] | 357.0[221.0,724.0] | 378.0[238.0,644.0] | 0.796 |
| ALT/AST ratio | 0.32[0.22,0.45] | 0.40[0.31,0.53] | 0.27[0.21,0.32] | **0.002** |
| Total protein, g/L | 59.73±5.55 | 60.02±4.64 | 59.40±6.40 | 0.693 |
| Albumin, g/L | 31.87±4.43 | 31.85±3.91 | 31.89±4.95 | 0.975 |
| Albumin/globulin ratio | 1.17±0.25 | 1.16±0.25 | 1.19±0.26 | 0.697 |
| TBIL, μmol/L | 7.9[5.3,10.3] | 7.9[5.1,10.3] | 8.3[5.5,10.2] | 0.669 |
| DBIL, μmol/L | 3.8[2.9,6.5] | 3.6[2.6,6.5] | 4.2[3.0,6.3] | 0.305 |
| ALP, U/L | 75.0[58.0,98.0] | 74.0[58.0,111.0] | 81.0[65.0,95.0] | 0.852 |
| γ-Glutamyl transferase,U/L | 38.0[22.0,82.0] | 40.0[24.0,82.0] | 34.0[21.0,71.0] | 0.397 |
| LDH, U/L | 926.0[634.0,1474.0] | 860.0[601.0,1420.0] | 975.0[681.0,1474.0] | 0.402 |
| Potassium,mmol/L | 4.10±0.51 | 4.17±0.59 | 4.04±0.38 | 0.365 |
| Sodium,mmol/L | 133.0[130.4,134.4] | 133.1[131.0,134.4] | 132.5[130.2,134.4] | 0.776 |
| Chlorine,mmol/L | 100.29[96.40,102.50] | 100.80[96.40,102.50] | 100.00[97.70,102.40] | 0.943 |
| Calcium,mmol/L | 1.95±0.15 | 1.98±0.11 | 1.91±0.18 | 0.084 |
| Urea, mmol/L | 6.00[4.66,9.00] | 5.20[4.20,6.98] | 6.40[5.30,11.51] | **0.044** |
| Creatinine, μmol/L | 89.0[68.0,117.0] | 89.0[68.0,99.0] | 102.0[72.0,134.0] | 0.199 |
| Urea, mmol/L | 266.0[210.0,333.0] | 254.0[210.0,329.0] | 287.0[211.3,355.8] | 0.397 |
| HCO3^−^, mmol/L | 18.99±3.50 | 19.32±3.23 | 18.61±3.75 | 0.473 |
| eGFR, mL/min/1.73m2 | 67.66±24.11 | 73.57±24.37 | 61.05±22.01 | 0.061 |
| Lactic acid,mmol/L | 1.74[1.27,2.20] | 1.66[1.21,2.14] | 1.76[1.47,2.36] | 0.176 |
| Glucose, mmol/L | 7.40[6.30,9.67] | 6.75[5.72,8.92] | 8.43[6.87,9.69] | 0.065 |
| Lipase, IU/L | 217.5[125.3,377.4] | 182.6[98.7,334.6] | 278.1[197.6,419.2] | **0.028** |
| Amylopsin, U/L | 96.0[62.0,147.0] | 88.0[51.0,108.0] | 102.0[80.0,147.0] | 0.051 |
| CK,U/L | 500.0[246.0,1371.0] | 456.0[246.0,1349.0] | 686.0[250.0,1371.0] | 0.782 |
| Triglyceride, mmol/L | 3.02[1.46,3.79] | 3.14[1.70,3.79] | 1.99[1.38,3.71] | 0.397 |
| Total cholesterol, mmol/L | 2.91±0.89 | 3.05±0.67 | 2.75±1.06 | 0.239 |
| **Coagulation markers** |  |  |  |  |
| PT,s | 13.30±1.52 | 13.00±1.45 | 13.64±1.52 | 0.127 |
| APTT,s | 62.4[54.7,85.7] | 59.3[50.9,70.4] | 64.9[55.8,97.9] | 0.193 |
| TT,s | 31.9[22.2,50.6] | 29.5[22.3,39.5] | 38.5[22.2,57.9] | 0.482 |
| Fibrinogen, g/L | 2.36[2.10,2.75] | 2.68[2.26,2.96] | 2.16[1.80,2.55] | **0.003** |
| D-dimer, μg/mL FEU | 6.08[3.74,12.71] | 5.72[2.54,11.62] | 7.10[4.70,13.43] | 0.144 |
| **Cardiac Markers** |  |  |  |  |
| Myoglobin,ng/mL | 189.5[109.2,567.6] | 142.5[103.0,390.1] | 381.2[153.0,710.1] | **0.029** |
| CK-MB,ng/mL | 3.1[1.3,7.5] | 3.0[1.0,7.5] | 4.7[2.0,7.5] | 0.262 |
| hs-cTnI,pg/mL | 72.6[29.4,211.3] | 45.0[24.5,140.9] | 115.9[68.0,274.1] | **0.019** |
| NT-proBNP,pg/mL | 514.4[199.7,1244.0] | 394.0[171.4,884.0] | 642.0[321.0,1244.0] | 0.193 |
| **Inflammatory indicators** |  |  |  |  |
| Ferritin, μg/L | 19733.0[7569.7,45124.0] | 17370.0[5725.3,40471.0] | 21294.0[11630.0,45124.0] | 0.402 |
| hsCRP, mg/L | 7.7[4.2,13.1] | 8.6[3.7,12.7] | 7.7[4.6,16.8] | 0.682 |
| PCT, ng/mL | 0.9[0.42,2.0] | 0.48[0.13,1.47] | 1.47[0.90,2.35] | **0.001** |
| **Cytokine indicators** |  |  |  |  |
| IL-6, pg/mL | 84.45[49.90,143.30] | 76.02[18.08,117.60] | 85.33[55.89,172.63] | 0.089 |
| IL-2R, U/mL | 1479.0[1138.0,2077.0] | 1516.0[1163.0,1930.0] | 1479.0[1138.0,2130.0] | 0.637 |
| IL-8, pg/mL | 51.0[25.6,128.0] | 36.2[20.4,84.4] | 69.2[40.9,128.0] | 0.125 |
| IL-10, pg/mL | 100.0[53.5,181.0] | 66.9[34.9,124.0] | 159.0[68.7,216.0] | **0.020** |
| IL-1β, pg/mL | 7.2[5.0,17.9] | 5.0[5.0,13.2] | 9.8[7.0,17.9] | **0.037** |
| TNF-α, pg/mL | 31.5[21.40,67.35] | 31.60[22.40,60.40] | 30.00[21.40,69.80] | 0.438 |
