# Supplementary material for: Unveiling fatal risk factors: Predicting hemophagocytic lymphohistiocytosis in SFTS patients
Source: PLoS Negl Trop Dis. 2025 Jun 24;19(6):e0013207. doi: 10.1371/journal.pntd.0013207 (PMC12186921; doi:10.1371/journal.pntd.0013207)
Supplement: S2 Table — (DOCX) [file pntd.0013207.s002.docx]

**Supplementary Table 2** ROC Curve Analysis for Predicting Death in SFTS Patients with HLH

| **Index** | **AUC (95%CI)** | **Sensitivity** | **Specificity** | **Youden index** | **Cut-off value** |
| --- | --- | --- | --- | --- | --- |
| Age,year | 0.804(0.670-0.928) | 0.760(0.560-1.000) | 0.714(0.362-0.937) | 0.474(0.359-0.770) | 64.000(56.000-68.000) |
| FIB,g/L | 0.741(0.616-0.884) | 0.893(0.471-1.000) | 0.600(0.452-0.920) | 0.493(0.282-0.718) | 2.230(2.230-2.810) |
| PCT, ng/mL | 0.761(0.629-0.876) | 0.760(0.555-1.000) | 0.714(0.481-0.969) | 0.474(0.349-0.675) | 0.900(0.440-2.190) |
| A-F-P | 0.903(0.821-0.973) | 0.880(0.731-1.000) | 0.821(0.688-0.971) | 0.701(0.586-0.902) | 0.345(0.221-0.791) |

A-F-P representing the combination of Age, Fib ( fibrinogen), and PCT (Procalcitonin).
