# Supplementary figures and images for: Unveiling fatal risk factors: Predicting hemophagocytic lymphohistiocytosis in SFTS patients
Source: PLoS Negl Trop Dis. 2025 Jun 24;19(6):e0013207. doi: 10.1371/journal.pntd.0013207 (PMC12186921; doi:10.1371/journal.pntd.0013207)

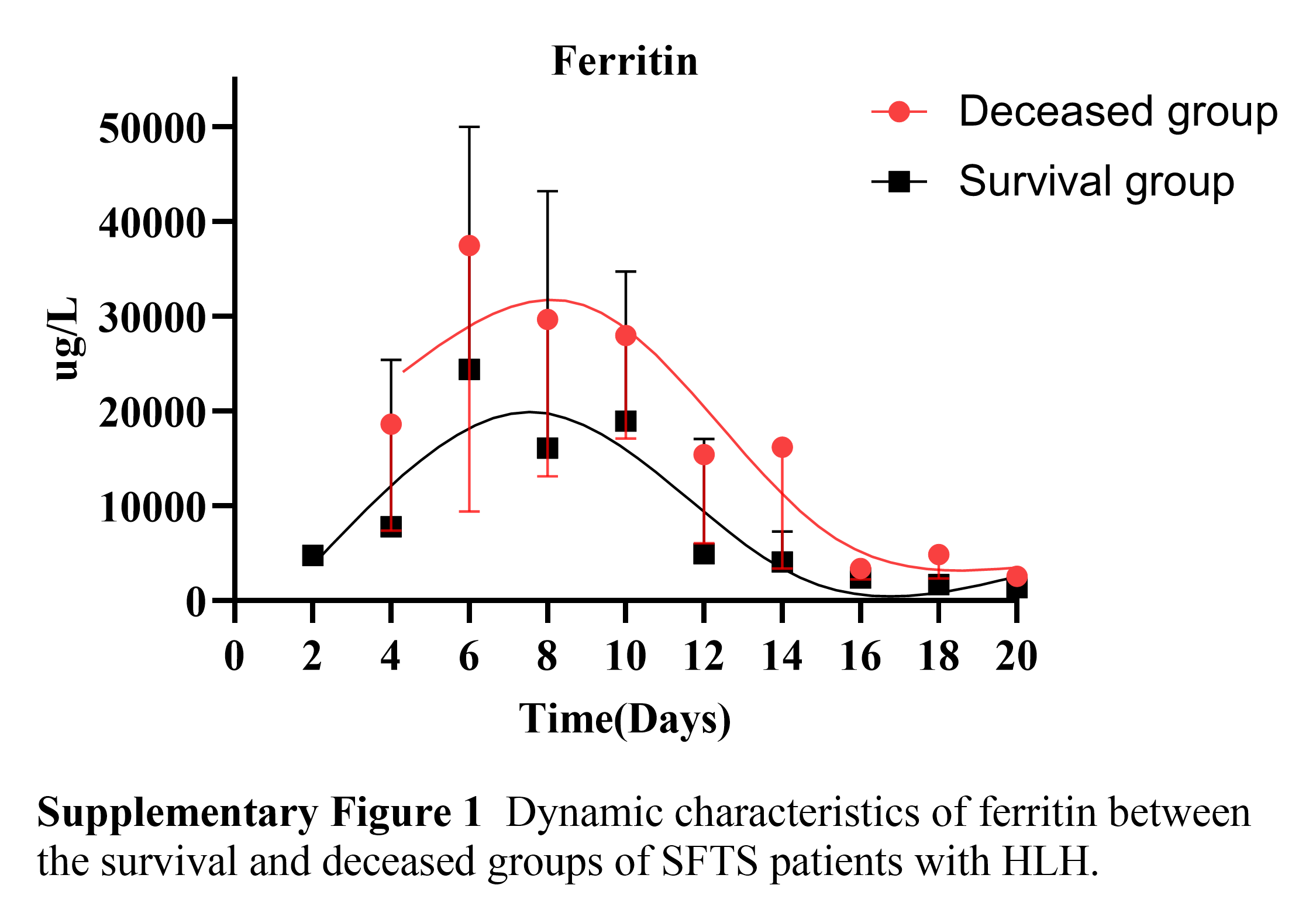

Supplement: S1 Fig — (TIF) [file pntd.0013207.s003.tif]
