# Supplementary material for: Unveiling fatal risk factors: Predicting hemophagocytic lymphohistiocytosis in SFTS patients
Source: PLoS Negl Trop Dis. 2025 Jun 24;19(6):e0013207. doi: 10.1371/journal.pntd.0013207 (PMC12186921; doi:10.1371/journal.pntd.0013207)
Supplement: Attachment — Submitted filename: PNTD-D-24-01931_reviewer_submitted.pdf [file pntd.0013207.s004.pdf]

Reviewer Recommendation and Comments for Manuscript Number PNTD-D-24-01931

Dear Editor,

I have reviewed the manuscript titled "Unveiling Fatal Complications: Predicting Hemophagocytic Lymphohistiocytosis (HLH) in SFTS Patients" and have provided my detailed feedback below. Overall, the study presents important insights into identifying early predictive and prognostic factors for patients with Severe Fever with Thrombocytopenia Syndrome (SFTS) and concurrent HLH, a rare and fatal complication. The study analyzed clinical and laboratory data from 272 SFTS patients using logistic regression to determine key factors associated with HLH and mortality. The findings of the study indicate that decreased platelet count, low ALT/AST ratio, elevated LDH, and increased DD levels are risk factors for HLH in SFTS patients. Furthermore, age, fibrinogen (FIB), and procalcitonin (PCT) were identified as independent mortality risk factors in patients with HLH. The combination of these factors effectively predicts patient prognosis, highlighting the importance of monitoring these indicators to guide clinical management and reduce mortality. The research also emphasizes the need for dynamic monitoring of FIB and PCT levels to track disease progression. While the study makes a valuable contribution to the field, I have identified several areas that require clarification, further analysis, or additional references. Below are my comments organized by section:

1. **Missing References**: The references after number 27 are missing, which prevents me from reviewing the demographic information of the patients in references [57, 71]. However, Table 1 includes the demographic details for these patients. Please ensure that the references after number 27 are included to complete the citation list.
2. **Sample Size Disparity**: Among the study participants, 53 were in the HLH group, and 219 were in the non-HLH group. The disparity in sample sizes between these two groups could impact the statistical power of the comparisons. Specifically:
    - **Smaller HLH Group**: A smaller sample size in the HLH group introduces greater variability, making it harder to detect true associations and increasing the risk of overfitting.
    - **Statistical Implications**: Logistic regression models may be affected by this imbalance, potentially leading to wide confidence intervals and reduced reliability of the conclusions.

    How to Address Potential Bias:

    - **Use Statistical Adjustments**: Techniques like propensity score matching (PMID: 9802183) can help mitigate the impact of unequal group sizes and reduce potential bias.
3. **Statistical Testing for Figure 3A and B**: It would be helpful for the authors to clarify whether statistical tests were applied to compare the levels of variables shown in Figure

3A and B. Clearer reporting of statistical analyses would enhance the interpretation of these figures.

4. **Discussion on Diarrhea and HLH Risk in SFTS Patients**: In the Discussion section (Lines 259-262), the authors report a discrepancy between their findings and those of Reference 15 regarding the role of diarrhea in the risk of mortality in SFTS patients. While the authors suggest that diarrhea may reduce the risk of HLH (OR = 0.434), Reference 15 indicates that diarrhea increases the risk of death. The authors attribute this difference to the shorter time from symptom onset to hospital admission, allowing for timely intravenous treatment.

   However, it is unclear whether the authors considered the duration and severity of diarrhea over the entire hospitalization period. A dynamic follow-up of diarrhea incidence and intensity could provide a more comprehensive understanding of its role in patient outcomes. For instance:

   - Diarrhea may have been more prevalent in the survival group at admission but could have worsened or intensified in the deceased group later in the hospitalization.
   - Prolonged or worsening diarrhea could be a marker of disease severity, rather than an independent protective or risk factor.

   To resolve this discrepancy, a longitudinal follow-up approach—including daily clinical assessments, laboratory monitoring (e.g., electrolytes, inflammatory markers), and statistical analyses (e.g., Kaplan-Meier survival curves or mixed-effects models)—could help clarify the role of diarrhea in patient prognosis. The authors should indicate whether such an analysis was performed or consider incorporating it into the study.

5. **Longitudinal Ferritin Trends**: Although ferritin was not found to be an independent risk factor in the multivariate analysis, it is referenced in other studies as a potential predictor of mortality in SFTS and HLH patients. A longitudinal analysis tracking ferritin levels throughout hospitalization (e.g., daily or weekly trends between survivors and non-survivors) could reveal temporal patterns that a single time-point measurement cannot capture. This additional analysis could enhance the study's insight into ferritin's role in patient prognosis.
6. **Cohort Enrollment and Geographic Variation**: The authors did not clarify whether the cohort was enrolled at the same time and whether the patients were from similar geographic regions (e.g., urban vs. rural). This distinction is important because location and timing (e.g., seasonal variations) can influence viral transmission and disease severity. Different geographic regions may also be associated with different strains or genotypes of the virus, leading to varied outcomes, including differences in mortality rates. Studies such as PMID: 38257828, PMID: 35603493, and PMID: 33953322 highlight how these factors can impact disease progression and outcomes. Clarifying these aspects would improve the external validity of the study and its relevance to real-world settings.
7. **Viral Genotypes and Association with Mortality**: The study does not address whether the viral genotype or strain (e.g., A, B, D, E, F) differs between the survival and deceased

groups. Analyzing whether specific viral genotypes are associated with mortality could provide valuable insights. Studies like PMID: 30136250 and PMID: 39530174 suggest that viral genetic variability can influence patient outcomes. The authors should consider whether the viral genotype correlates with viral load or mortality risk, as this could enrich the study's findings and provide more precise recommendations for treatment.

8. **Viral Load and Clinical Risk Factors**: The authors did not investigate whether the viral load correlates with any of the clinical markers identified as risk factors (e.g., age, FIB, PCT, ALT/AST ratio, LDH, DD) or interacts with the HLH vs. non-HLH groups. Analyzing the relationship between viral load and these clinical markers could reveal additional insights into mortality risk. Exploring this relationship could potentially strengthen the study's conclusions regarding the viral factors influencing SFTS patient prognosis and improve understanding of how viral load interacts with other risk factors.

Conclusion: This manuscript presents important findings on the role of various clinical markers in predicting outcomes for SFTS patients. While the study is well-designed and informative, the issues raised above should be addressed to enhance the robustness and clarity of the findings. In particular, the authors should consider addressing the sample size disparity, incorporating longitudinal analyses of key markers like ferritin and diarrhea, and providing further details on cohort enrollment and geographic variation. The incorporation of viral genotype data and the exploration of viral load interactions with clinical markers would significantly enrich the manuscript and improve its contribution to the field.

I recommend that the authors address these concerns and resubmit the manuscript after making the necessary revisions.
