# Supplementary material for: Unveiling fatal risk factors: Predicting hemophagocytic lymphohistiocytosis in SFTS patients
Source: PLoS Negl Trop Dis. 2025 Jun 24;19(6):e0013207. doi: 10.1371/journal.pntd.0013207 (PMC12186921; doi:10.1371/journal.pntd.0013207)
Supplement: Attachment — Submitted filename: Response to Reviewers.docx [file pntd.0013207.s005.docx]

Dear editor,

We would like to take this opportunity again to thank the editors and reviewers for their very constructive suggestions. These comments and suggestions helped us a lot on guiding the revisions in every detail, which makes our manuscript more sound and creditable. On the other hand, these processes benefited us on developing our abilities in rigorous thinking and scientific writing, which we also appreciate very much.

Now we have performed additional analyses, made necessary changes of both text and figures to acknowledge every issue and concern from all the reviewers. As a result, we believe that the manuscript is greatly strengthened and ready for publication. Please see below for our detailed responses. The comments from the decision letter are copied in blue and italic, while our responses are in black.

**Methods:**

*1.The objectives for this paper – identifying predictive biomarkers for HLH and markers for. HLH mortality – are clearly stated. There are no citations provided for the methods section, however, and therefore it is difficult to ascertain whether the methods used were appropriate or if they were novel to the manuscript.Additionally, in 102, the author refers to a “big data platform”, please provide the specific data platform. The n-size for the total population and the population that were HLH positive appear appropriate to address the objective of this manuscript.*

**Answer:**We sincerely appreciate the reviewers’ insightful comments regarding the absence of references in the Methods section. We fully acknowledge that incorporating appropriate citations would further strengthen the methodological robustness of our study.

Although there have been previous studies reporting the occurrence of HLH in SFTS patients ^[1-4]^, these were mainly isolated case reports without any correlation analysis. Secondly,This exploratory study aims to identify predictive biomarkers for secondary HLH (hemophagocytic lymphohistiocytosis) in SFTS patients, as well as mortality-associated markers, by analyzing demographic characteristics, clinical presentations upon admission, and laboratory parameters. The hematological markers, biochemical indices, inflammatory markers, cardiac markers, coagulation parameters, and cytokines listed in the manuscript represent laboratory data specifically collected from SFTS patients in this investigation. As such, these parameters do not require citation of external references.

Concerning the "HLH-2004 diagnostic criteria," the manuscript explicitly cites the following reference: **[12] Henter JI, Horne A, Aricó M, et al. HLH-2004: Diagnostic and therapeutic guidelines for hemophagocytic lymphohistiocytosis.** Pediatr Blood Cancer. **2007;48(2):124-131. doi:10.1002/pbc.21039**

With respect to the statistical methods employed, only fundamental analytical techniques—widely recognized as standard practice in the field—were utilized. Therefore, no additional references were provided for this section.

Furthermore, The paper mentions that the "Big Data Platform" serves as the electronic medical record data center of the research institution. This platform enables comprehensive retrieval of all patient case information, while strictly encrypting and desensitizing data involving patient privacy. To enhance clarity, we have revised this term to "Medical Data Retrieval and Application Platform" in the revised manuscript.

**Results:**
1. *The analysis presented matches the analysis plan. The results are clear and completely presented. The figures do appear slightly blurry, and this should be fixed before publication, but the content of the figures is clear.*

**Answer:**We sincerely appreciate the reviewers’ valuable comments regarding the image resolution. We apologize for any inconvenience caused during the review process.

We have carefully re-examined all submitted figures and confirmed that they fully comply with the journal's requirements. The submitted manuscript's PDF version may experience image compression and reduced clarity during the submission system's file conversion process. The original high-resolution image files are available for direct download if needed.

**Conclusions:**
*1. The conclusions drawn are clearly supported by the data presented. The authors additionally contextualize their findings within the larger body of research or attempt to explain how their work disagrees with prior studies (i.e., the diarrhea results). The authors do discuss some limitations to their work, and while some are very clear, such as markers that could have been studied, some are far too vague. Specifically, the authors should expand on the phrase “influencing factors may not have been included”. Overall, this manuscript has significant public health relevance, as it provides critical diagnostic criteria and relationships between them to predict HLH”.*

**Answer:**We sincerely appreciate the reviewers' recognition of the data support and academic value of our study. We have carefully addressed all suggestions by supplementing potentially overlooked influencing factors and analyzing their impact on our conclusions. The main points are summarized as follows:

① **Diagnostic Limitations**: Although the HLH-2004 criteria ^[5]^ remain the most widely used standard for HLH diagnosis, they have certain limitations. Parameters such as hemophagocytosis, serum NK cell activity, and sCD25 concentration are not routinely measured, which may lead to underdiagnosis. Additionally, variability in physicians' diagnostic criteria and documentation practices may introduce potential bias. Some SFTS patients, particularly those with complex presentations or in early stages of the disease, might not meet all diagnostic criteria, resulting in missed diagnoses.

② **Retrospective Data Constraints**: The retrospective nature of this study may not capture all relevant variables. For instance, the duration and severity of diarrhea during hospitalization were not consistently recorded, which could influence outcome assessments.

③ **Sample Size Disparity**: The unequal sample sizes between SFTS patients with and without HLH might affect the statistical power of our comparisons. Although we adjusted for known confounders using multivariate regression, residual confounding may persist. Future studies with larger cohorts are needed to address this limitation.

④ **Dynamic Factors**: Retrospective data may not fully account for dynamic variables such as viral load fluctuations ^[6-7]^ and individual immune status ^[8-9]^, which could impact HLH development and prognosis in SFTS patients. Prospective studies are warranted to better control for these factors.

⑤ **Regional and Viral Strain Variability**: Geographic differences in viral strains and genotypes may influence SFTS outcomes ^[10]^. Whether specific genotypes affect HLH incidence or prognosis in SFTS patients remains unclear and requires further investigation.

All modifications in the revised manuscript have been clearly marked to facilitate your review. We are deeply grateful for your expert guidance, which has significantly enhanced the quality of our work.

**Editorial and Data Presentation Modifications：**

1.*What is the full name of APTT in line 113?*

*2.It is necessary to correct the usage of spacing in the manuscript, as there were many errors (line 114, 121, 123, 125, 127, 151~155, Table 1~3, 166, 169, 171, 190~195, and etc…). For example: in table 1 '(n=272)', the letter 'n' should be italicized, and there should be a single space between each character like ‘(n = 272)’.*

*3. In general, the paper should be reviewed for grammatical errors, such as in Line 90, which should say “We gathered hospitalized SFTS patients…”*

**Answer:**We thank the reviewers for their suggestions on the writing issues in this manuscript. After rechecking, we have corrected the English grammar, word spelling, and formatting issues in the manuscript. All modifications will be highlighted in yellow in the revised version for your review. We appreciate your help in improving the quality of this paper.

**Summary and General Comments：**

*1.The authors of this study provide insufficient explanation regarding the changes in the blood coagulation mechanism associated with increased procalcitonin (PCT) levels. The hypothesis that PCT elevation worsens the prognosis of SFTS patients by enhancing systemic inflammation lacks sufficient supporting evidence. Providing a discussion on the correlation between PCT and disseminated intravascular coagulation (DIC) would strengthen the argument. Additional explanation in the Discussion section is essential.*

**Answer:**Thank you for your valuable suggestions on this manuscript. We have supplemented and improved the Discussion section according to your recommendations, with specific modifications as follows:

(1) In the Discussion section, we further clarified the correlation between elevated PCT levels and the prognosis of SFTS patients, as well as its association with HLH comorbidity. Research indicates that elevated PCT levels are significantly associated with increased mortality rates in patients with SFTS, particularly during the acute phase of the illness. Variations in PCT levels may thus serve as a crucial prognostic marker for evaluating patient outcomes ^[11-12]^. HLH is a severe hyperinflammatory syndrome ^[13]^. SFTS patients complicated by HLH exhibit significantly higher ferritin and PCT levels compared to non-HLH patients. This observation suggests that ferritin and PCT may hold critical clinical significance in assessing disease severity and prognosis.
(2) We added a discussion on the relationship between PCT levels and coagulation dysfunction in SFTS patients. Beyond systemic inflammation, PCT may exacerbate the clinical course of SFTS by activating coagulation pathways. Research indicates that coagulation dysfunction in SFTS patients is closely linked to elevated PCT levels, potentially due to PCT promoting the release of coagulation factors during inflammatory responses, thereby triggering coagulation activation ^[14]^. In studies of COVID-19 patients, serum PCT levels were found to be positively correlated with disease severity and coagulopathy. Notably, elevated PCT levels in severe infections such as COVID-19 may indicate the presence of disseminated intravascular coagulation (DIC) ^[15-16]^. Furthermore, research has demonstrated that SFTS-associated DIC patients exhibit significantly higher levels of inflammatory markers, including interleukin-6 (IL-6) and C-reactive protein (CRP). These biomarkers, similar to PCT, may play critical roles in the pathogenesis of DIC^[17]^. Therefore, elevated PCT levels in SFTS patients may reflect both disease severity and coagulation dysfunction.

All modifications in the revised manuscript have been clearly marked to facilitate your review. We sincerely appreciate your contributions to enhancing the quality of this work!

*2.In Table 1&2, which compares non-HLH and HLH patients, the HLH-classified group shows increased APTT and TT, decreased fibrinogen, and increased D-dimer, all indicating reduced blood coagulation activity. This could suggest that SFTS patients diagnosed with HLH may have impaired coagulation function, potentially leading to a lower incidence of DIC. However, as the authors mentioned in lines 268–269, critical SFTS patients are at high risk due to progression to DIC and MODS, which seems contradictory. Furthermore, as seen in the outcome data, patients classified as HLH show a significantly higher mortality rate. The authors appear to have skipped an in-depth discussion of this discrepancy and moved directly to analyzing risk factors in HLH-classified SFTS patients. A more thorough discussion of Table 1&2 is essential to provide a clearer interpretation of these findings.*

**Answer:** Thank you for the reviewer’s insightful feedback on the data presented in Tables 1 and 2 of our manuscript. We provide further clarification and supporting evidence below:

(1) Higher Mortality in SFTS Patients Complicated by HLH：In an analysis of 18 patients with SFTS, 33.3% developed complications from HLH . The mortality rate for SFTS patients with HLH was 75%, in contrast to a significantly lower mortality rate among those without HLH ^[3]^. These findings are consistent with our study, highlighting that SFTS patients with HLH complications experience a markedly increased risk of mortality.
(2) The seemingly contradictory phenomenon between the coagulation indexes of patients with HLH and the risk of DIC：SFTS patients in the early stages typically present with fever, thrombocytopenia, and bleeding tendencies (e.g., ecchymosis, gastrointestinal bleeding), which are attributed to direct viral damage to endothelial cells and megakaryocytes, leading to thrombocytopenia and increased vascular permeability ^[8]^. As the disease progresses, some patients develop DIC , resulting in multiple organ failure and significantly elevated mortality.SFTS patients complicated by HLH at admission often exhibit prolonged activated partial thromboplastin time (APTT) and thrombin time (TT), as well as reduced fibrinogen levels. These coagulation abnormalities may stem from systemic inflammatory responses and immune hyperactivation. Studies suggest that elevated levels of anti-endothelial cell antibodies (AECA) and anti-phospholipid antibodies (aPLs) in SFTS patients correlate with coagulation dysfunction, including prolonged APTT and TT ^[18]^. Additionally, SFTS patients demonstrate markedly higher levels of inflammatory cytokines and chemokines (e.g., IL-1β, IL-6, IL-8, CCL2, CXCL10) compared to healthy controls, and these aberrant immune responses may further contribute to coagulation dysregulation ^[18]^.In HLH patients, coagulation abnormalities may undergo dynamic evolution. During the early phase, excessive activation of macrophages by inflammatory cytokines drives hyperfibrinolysis (manifested as elevated D-dimer and reduced fibrinogen), resulting in a hypocoagulable state. However, as endothelial injury worsens, this may transition to a hypercoagulable phase characterized by microvascular thrombosis, ultimately progressing to DIC ^[19]^. Thus, the observed coagulation parameters in HLH patients do not contradict DIC risk but rather reflect the dynamic nature of the disease.
(3) Supplement the analysis of blood routine indicators, liver function markers, coagulation markers, inflammatory markers, and cytokine markers with significant differences in Table 2：HLH patients typically exhibit pancytopenia due to pancytopenia due to cytokine storm, macrophage activation, bone marrow suppression, and increased hemophagocytosis ^[13,20,21]^,which explains why SFTS patients complicated by HLH demonstrate significantly lower leukocyte, lymphocyte, monocyte, and platelet counts compared to non-HLH cases;

In SFTS patients, elevated ALT, AST, and a reduced ALT/AST ratio are widely used to assess hepatic injury. A decreased ALT/AST ratio may indicate severe liver damage and is associated with poorer prognosis in SFTS patients ^[12]^. Additionally, elevated LDH, reflecting the extent of tissue injury and cellular destruction, has been identified as a marker of adverse outcomes in SFTS ^[22]^. Notably, SFTS patients with HLH exhibit higher ALT, AST, and LDH levels, along with a lower ALT/AST ratio, all of which collectively signal worse prognosis;

In patients with HLH upon admission, the elevation of APTT, TT and DD, as well as the decrease of FIB, may be related to the inflammatory response and excessive activation of the immune system in SFTS patients ^[18]^. In SFTS patients complicated with HLH, the coagulation abnormalities may present dynamic evolution characteristics: in the early stage, the coagulation indexes are mainly characterized by hypocoagulation; however, with the aggravation of endothelial injury, microvascular thrombosis may occur in the later stage (hypercoagulation stage), ultimately progressing to DIC ^[19]^ ;

Ferritin and PCT, as inflammatory biomarkers, demonstrate significant clinical value in the early prognostic assessment of SFTS patients ^[11]^. Studies have confirmed that elevated PCT levels are significantly associated with increased mortality in SFTS patients, particularly during the acute phase of the disease, where changes in PCT levels can serve as an important indicator for evaluating prognosis ^[11-12]^. Notably, extremely elevated ferritin levels in HLH patients have been clearly identified as a predictor of disease severity and poor prognosis ^[23]^. The results of this study indicate that SFTS patients complicated with HLH have significantly higher ferritin and PCT levels compared to non-HLH patients, suggesting that ferritin and PCT may hold important clinical significance in disease severity and prognosis.

Studies have found that elevated levels of serum IL-6 and IL-10 at admission are independent risk factors for in-hospital mortality in SFTS patients ^[24]^. It is worth noting that when SFTS is complicated with HLH, the synergistic elevation of IL-6, IL-2R, and IL-10 can exacerbate cytokine storm ^[25]^. Therefore, monitoring cytokines during the acute phase may guide immunomodulatory therapy to suppress excessive inflammatory responses and assess patient prognosis.

All modifications in the revised manuscript have been clearly marked to facilitate your review. We sincerely appreciate your invaluable feedback in enhancing the scientific rigor of this work.

*3.Based on the title of the paper, one might anticipate that the research focuses on distinguishing HLH among SFTS patients. However, the precise final conclusion of this study is the identification of important factors for assessing the risk of SFTS patients classified as HLH. Therefore, I believe it is necessary to revise the title.*

**Answer:** We express our sincere gratitude for your thorough review and invaluable feedback on our study. Your expert critique concerning the alignment between the title and the manuscript content is exceptionally insightful, and we fully agree with your perspective. In response to your recommendation, we have revised the title to: "**Unveiling Fatal Risk Factors: Predicting Hemophagocytic Lymphohistiocytosis in SFTS Patients.**" This revised title more accurately encapsulates the study’s focus on identifying critical prognostic indicators for the development of HLH in SFTS patients. We extend our thanks once again for your guidance in enhancing the precision and clarity of our work.

4. *However, this paper is not without issues, some of which are major and some minor, that must be addressed before it is accepted. The primary issue that requires significant revisions is that the authors failed to provide any citations within the methods section. I am unable to determine whether the methods used are novel or adapted from existing work due to the absence of citations, which makes it impossible to evaluate the study's originality and reproducibility. The secondary issue is that the limitation section, particularly when discussing confounding factors, is too vague to be interpreted appropriately .*

**Answer:**Thank you for your meticulous review and invaluable feedback on our manuscript. We have carefully addressed your concerns and provide the following clarifications and revisions:

(1) Clarification on the Lack of References in the Methods Section：Although there have been previous studies reporting the occurrence of HLH in SFTS patients ^[1-4]^, these were mainly isolated case reports without any correlation analysis. Secondly,This exploratory study aims to identify predictive biomarkers for secondary HLH in SFTS patients, as well as mortality-associated markers, by analyzing demographic characteristics, clinical presentations upon admission, and laboratory parameters. The hematological markers, biochemical indices, inflammatory markers, cardiac markers, coagulation parameters, and cytokines listed in the manuscript represent laboratory data specifically collected from SFTS patients in this investigation. As such, these parameters do not require citation of external references.Concerning the "HLH-2004 diagnostic criteria," the manuscript explicitly cites the following reference: **[12] Henter JI, Horne A, Aricó M, et al. HLH-2004: Diagnostic and therapeutic guidelines for hemophagocytic lymphohistiocytosis. Pediatr Blood Cancer. 2007;48(2):124-131. doi:10.1002/pbc.21039.** With respect to the statistical methods employed, only fundamental analytical techniques—widely recognized as standard practice in the field—were utilized. Therefore, no additional references were provided for this section.
(2) Regarding the vague description of limitations and confounding factors, we will rewrite the section on limitations, listing the potential confounding factors that may affect the results one by one and analyzing the impact of these factors on the conclusions.**：**① Diagnostic Limitations: Although the HLH-2004 criteria ^[5]^ remain the most widely used standard for HLH diagnosis, they have certain limitations. Parameters such as hemophagocytosis, serum NK cell activity, and sCD25 concentration are not routinely measured, which may lead to underdiagnosis. Additionally, variability in physicians' diagnostic criteria and documentation practices may introduce potential bias. Some SFTS patients, particularly those with complex presentations or in early stages of the disease, might not meet all diagnostic criteria, resulting in missed diagnoses.② Retrospective Data Constraints: The retrospective nature of this study may not capture all relevant variables. For instance, the duration and severity of diarrhea during hospitalization were not consistently recorded, which could influence outcome assessments.③ Sample Size Disparity: The unequal sample sizes between SFTS patients with and without HLH might affect the statistical power of our comparisons. Although we adjusted for known confounders using multivariate regression, residual confounding may persist. Future studies with larger cohorts are needed to address this limitation.④ Dynamic Factors: Retrospective data may not fully account for dynamic variables such as viral load fluctuations ^[6-7]^ and individual immune status ^[8-9]^, which could impact HLH development and prognosis in SFTS patients. Prospective studies are warranted to better control for these factors.⑤ Regional and Viral Strain Variability: Geographic differences in viral strains and genotypes may influence SFTS outcomes ^[10]^. Whether specific genotypes affect HLH incidence or prognosis in SFTS patients remains unclear and requires further investigation.

All modifications in the revised manuscript have been clearly marked to facilitate your review. We are deeply grateful for your expert guidance, which has significantly enhanced the quality of our work.

**Reviewer Recommendation and Comments for the PDF Document in the Email Attachment**

1.*Missing References: The references after number 27 are missing, which prevents me from reviewing the demographic information of the patients in references [57, 71]. However, Table 1 includes the demographic details for these patients. Please ensure that the references after number 27 are included to complete the citation list.*

**Answer:** We extend our gratitude to the reviewer for highlighting the issue with the phrase “The median age was 65 [57, 71] years.” Upon thorough examination, we identified that the notation “[57, 71]” was inaccurately employed in the manuscript. It was intended to represent the interquartile range (IQR) of the median age of the patients, rather than a reference citation. The text has been amended accordingly to read: “The median age was 65 (IQR, 57–71) years.”

*2 .Sample Size Disparity: Among the study participants, 53 were in the HLH group, and 219 were in the non-HLH group. The disparity in sample sizes between these two groups could impact the statistical power of the comparisons. Specifically:Smaller HLH Group: A smaller sample size in the HLH group introduces greater variability, making it harder to detect true associations and increasing the risk of overfitting；Statistical Implications: Logistic regression models may be affected by this imbalance, potentially leading to wide confidence intervals and reduced reliability of the conclusions.*

**Answer:** Thank you to the reviewer for raising the important issue regarding the difference in sample size between the HLH group (n=53) and the non-HLH group (n=219). We fully agree that this imbalance may affect the statistical results. However, after statistical calculations, we concluded that the impact of this imbalance on the results is still acceptable. As the reviewer mentioned, the small sample size of the HLH group may lead to reduced statistical power, and this discrepancy reflects the rarity of SFTS patients with concurrent HLH in the real world. A study that collected data from 4,567 SFTS patients found only 327 cases with HLH (7.16%) ^[26]^, which also indicates that SFTS patients with HLH are relatively few. Despite this limitation, we still chose to include all available HLH cases to ensure the clinical significance of our findings. To verify the impact of the sample differences, we have taken the following steps:

(1) Goodness-of-fit test for logistic regression: Hosmer-Lemeshow Test *χ*2=4.467, *p*=0.813>0.05. The model fits well.

2.Prediction accuracy was assessed using the ROC curve ^[27]^, with the model's ROC AUC = 0.795 > 0.7.The result is shown in the figure below. ( AUC > 0.7 is acceptable, and aAUC > 0.8 is considered excellent) ^[28]^.

3. We conducted a 1:2 propensity score matching (53 cases in the HLH group and 106 cases in the non-HLH group) and performed univariate and multivariate regression analyses again, obtaining similar results. The results are shown in the table below:

| **Parameters** | **Univariate** | | |  | **Multivariate** | | |
| --- | --- | --- | --- | --- | --- | --- | --- |
|  | **OR** | **95%CI** | ***P*** |  | **OR** | **95%CI** | ***P*** |
| Diarrhea | 0.592 | [0.287,1.224] | 0.157 |  | 0.431 | [0.193,0.964] | **0.040** |
| Platelet | 0.968 | [0.95,0.986] | 0.001 |  | 0.970 | [0.948,0.993] | **0.010** |
| ALT/AST ratio | 0.16 | [0.028,0.924] | 0.041 |  | 0.022 | [0.001,0.641] | **0.026** |
| APTT | 1.018 | [1.004,1.033] | 0.013 |  | 1.004 | [0.978,1.029] | 0.784 |
| Fibrinogen | 0.706 | [0.427,1.166] | 0.174 |  | 0.911 | [0.575,1.443] | 0.692 |
| DD | 1.094 | [1.039,1.152] | 0.001 |  | 1.069 | [1.011,1.132] | **0.020** |
| Time from onset to admission | 0.937 | [0.846,1.037] | 0.210 |  | 0.923 | [0.819,1.041] | 0.191 |
| LDH | 1.001 | [1.0,1.002] | 0.001 |  | 1.001 | [1.000,1.002] | **0.030** |
| Ferritin | 1.0 | [1.0,1.0] | 0.000 |  | 1.000 | [1.000,1.000] | **0.007** |
| IL-10 | 1.002 | [1.0,1.004] | 0.092 |  | 1.000 | [0.997,1.003] | 0.990 |

Research indicates that when the sample size is small, particularly when the treatment group comprises fewer than 100 cases, the quality of propensity score matching may be compromised. It is advisable to employ alternative methods or increase the sample size ^[29]^. Given that the sample size for the HLH group in our study is also fewer than 100 cases, there is potential for bias in the results. Consequently, we have addressed the issue of sample size in the 'Limitations' section of the original manuscript and recommended that future research expand the HLH cohort to validate the findings. We express our gratitude to the reviewer for their insightful suggestions, which have substantially enhanced the rigor of our study.

*3.Statistical Testing for Figure 3A and B: It would be helpful for the authors to clarify whether statistical tests were applied to compare the levels of variables shown in Figure 3A and B. Clearer reporting of statistical analyses would enhance the interpretation of these figures.*

**Answer:** Thank you to the reviewer for the professional suggestions. Figure 3 has now been updated to include statistical tests for the levels of the variables, with significance markers added and corresponding explanations provided in the figure legend.

4. *Discussion on Diarrhea and HLH Risk in SFTS Patients: In the Discussion section (Lines 259-262), the authors report a discrepancy between their findings and those of Reference 15 regarding the role of diarrhea in the risk of mortality in SFTS patients. While the authors suggest that diarrhea may reduce the risk of HLH (OR = 0.434), Reference 15 indicates that diarrhea increases the risk of death. The authors attribute this difference to the shorter time from symptom onset to hospital admission, allowing for timely intravenous treatment.However, it is unclear whether the authors considered the duration and severity of diarrhea over the entire hospitalization period. A dynamic follow-up of diarrhea incidence and intensity could provide a more comprehensive understanding of its role in patient outcomes.*

**Answer:** Thank you to the reviewer for the valuable suggestions regarding diarrhea and the risk of HLH in SFTS patients. Although some studies have reported that diarrhea increases the risk of death in SFTS patients ^[30]^, other studies have found that diarrhea is more common in SFTS patients who survive ^[31]^. Therefore, the role of diarrhea remains controversial in research. In our study, when comparing SFTS patients with and without HLH, we found that diarrhea was associated with a reduced risk of HLH. Further analysis of SFTS patients with HLH revealed no differences in diarrhea symptoms between the survival and death groups at admission. Additionally, the statistical results indicate that patients with concurrent HLH have a shorter interval from symptom onset to hospital admission. Thus, we speculate that the timely implementation of intravenous therapy may lead to the different associations between diarrhea and mortality.We concur with the reviewer's recommendation to dynamically monitor the incidence and severity of diarrhea. Limiting the assessment to the presence of diarrhea at the time of admission may result in an underestimation of its prognostic significance. Regrettably, due to the retrospective and exploratory nature of this study, we were unable to systematically document the duration and severity of diarrhea throughout the entire hospitalization period. We hope to further investigate the correlation between diarrhea and the occurrence of HLH in SFTS patients through prospective studies in the future. Thank you once again for the valuable suggestions from the reviewer.

5. *Longitudinal Ferritin Trends: Although* *ferritin was not found to be an independent risk factor in the multivariate analysis, it is referenced in other studies as a potential predictor of mortality in SFTS and HLH patients. A longitudinal analysis tracking ferritin levels throughout hospitalization (e.g., daily or weekly trends between survivors and nonsurvivors) could reveal temporal patterns that a single time-point measurement cannot capture. This additional analysis could enhance the study's insight into ferritin’s role in patient prognosis.*

**Answer:** Thank you to the reviewer for the valuable suggestions. Compared with non-HLH patients, patients with HLH had significantly higher ferritin levels at admission, which is consistent with other studies suggesting that it may indicate the risk of mortality in patients with SFTS and HLH ^[11, 32]^. In our study, when analyzing the prognosis of SFTS patients with HLH, we found that ferritin was not an independent risk factor for patient mortality. Therefore, we did not continue with the longitudinal analysis of ferritin levels. Following the reviewer's suggestion, we have conducted a supplementary analysis of the dynamic changes in ferritin levels during the hospital stay for patients with HLH (the results are shown in the figure below). Regrettably, based on the existing data, we did not observe differences in the sequential dynamic changes between the two groups. Since our study included only 53 patients with HLH, perhaps a larger number of cases is needed to further investigate the impact of ferritin dynamics on patient prognosis.

6. *Cohort Enrollment and Geographic Variation: The authors did not clarify whether the cohort was enrolled at the same time and whether the patients were from similar geographic regions (e.g., urban vs. rural). This distinction is important because location and timing (e.g., seasonal variations) can influence viral transmission and disease severity. Different geographic regions may also be associated with different strains or genotypes of the virus, leading to varied outcomes, including differences in mortality rates. Studies such as PMID: 38257828, PMID: 35603493, and PMID: 33953322 highlight how these factors can impact disease progression and outcomes. Clarifying these aspects would improve the external validity of the study and its relevance to realworld settings.*

**Answer:** Thank you to the reviewer for the suggestions regarding the enrollment period and geographic differences. These suggestions will greatly enhance the applicability of our study. Studies have shown that SFTS patients are mainly distributed in rural areas of mountainous and hilly regions, with cases occurring throughout the year but peaking in spring and summer ^[30]^. We categorized the 272 enrolled SFTS patients into two groups based on the season of enrollment: the spring - summer group (154 cases) and the autumn - winter group (118 cases). Additionally, we divided them into the rural group (234 cases) and the urban group (38 cases) based on geography. Statistical results indicated that there were no differences in the distribution of seasons or geographic locations between the HLH group and the non-HLH group (*P* > 0.05). Similarly, no differences were found in the distribution of seasons or geographic locations between the survival and death groups among HLH patients (*P* > 0.05). The specific results have been supplemented in the tables of the manuscript.

Different geographic regions may harbor different viral strains or genotypes ^[33-35]^, which could influence the prognosis of SFTS patients. However, whether different viral strains or genotypes affect the risk of HLH complications in SFTS patients still needs further exploration. We will supplement the statistics on the season and geographic location of disease onset in the revised manuscript and include relevant content in the discussion of limitations.

7. *Viral Genotypes and Association with Mortality: The study does not address whether the viral genotype or strain (e.g., A, B, D, E, F) differs between the survival and deceased groups. Analyzing whether specific viral genotypes are associated with mortality could provide valuable insights. Studies like PMID: 30136250 and PMID: 39530174 suggest that viral genetic variability can influence patient outcomes. The authors should consider whether the viral genotype correlates with viral load or mortality risk, as this could enrich the study’s findings and provide more precise recommendations for treatment.*

**Answer:** We express our gratitude to the reviewer for their insightful suggestions. The proposal to investigate the association between viral genotypes and mortality introduces a promising new avenue for our research. However, as this study is a retrospective case analysis, viral genotype testing was not conducted during the patients' hospitalization. Consequently, we are unable to evaluate whether different viral genotypes or strains influence the incidence of HLH in patients with SFTS or affect the prognosis of those with HLH. We will address this limitation in the discussion section of the manuscript. We sincerely appreciate the reviewer's professional advice, which significantly contributes to the scientific rigor and clinical relevance of our study. A discussion on the potential impact of viral genotypes will be included in the limitations section of the paper.

8. *Viral Load and Clinical Risk Factors: The authors did not investigate whether the viral load correlates with any of the clinical markers identified as risk factors (e.g., age, FIB, PCT, ALT/AST ratio, LDH, DD) or interacts with the HLH vs. non-HLH groups. Analyzing the relationship between viral load and these clinical markers could reveal additional insights into mortality risk. Exploring this relationship could potentially strengthen the study’s conclusions regarding the viral factors influencing SFTS patient prognosis and improve understanding of how viral load interacts with other risk factors.*

**Answer:**Thank you to the reviewer for the valuable feedback. We fully agree that analyzing the correlation between viral load and clinical markers may provide deeper insights into the prognostic mechanisms of SFTS patients. Previous studies have suggested that viral load in SFTS patients may be associated with disease severity ^[36]^, and that it is correlated with FIB, PCT, ALT/AST ratio, LDH, DD, etc.^[30]^. Nonetheless, the influence of viral load on the incidence of HLH in SFTS patients and its impact on the prognosis of HLH patients remain to be elucidated. Due to the retrospective design of this study, comprehensive viral load data were not available for all patients, precluding an analysis of the correlation between viral load and HLH occurrence. We plan to undertake prospective studies to systematically collect paired data on viral load and dynamic clinical indicators, thereby enabling a more thorough analysis of the relationship between viral load and HLH occurrence. We reiterate our appreciation to the reviewer for their constructive suggestions, which are instrumental in enhancing the quality of this study.

**REFERENCES**

1. Kim KH, Lee MJ, Ko MK, Lee EY, Yi J. Severe Fever with Thrombocytopenia Syndrome Patients with Hemophagocytic Lymphohistiocytosis Retrospectively Identified in Korea, 2008-2013. J Korean Med Sci. 2018;33(50):e319. doi:10.3346/jkms.2018.33.e319
2. Kitao A, Ieki R, Takatsu H, et al. Severe fever with thrombocytopenia syndrome presenting as hemophagocytic syndrome: two case reports. Springerplus. 2016;5:361. doi:10.1186/s40064-016-2010-2
3. Jung IY, Ahn K, Kim J, et al. Higher Fatality for Severe Fever with Thrombocytopenia Syndrome Complicated by Hemophagocytic Lymphohistiocytosis. Yonsei Med J. 2019;60(6):592-596. doi:10.3349/ymj.2019.60.6.592
4. Nakano A, Ogawa H, Nakanishi Y, et al. Hemophagocytic Lymphohistiocytosis in a Fatal Case of Severe Fever with Thrombocytopenia Syndrome. Intern Med. 2017;56(12):1597-1602. doi:10.2169/internalmedicine.56.6904
5. Henter JI, Horne A, Aricó M, et al. HLH-2004: Diagnostic and therapeutic guidelines for hemophagocytic lymphohistiocytosis. Pediatr Blood Cancer. 2007;48(2):124-131. doi:10.1002/pbc.21039。
6. Kwon JS, Kim JY, Jang CY, et al. Effect of Severe Fever With Thrombocytopenia Syndrome Virus Genotype on Disease Severity, Viral Load, and Cytokines in South Korea. Open Forum Infect Dis. 2024;11(9):ofae508. doi:10.1093/ofid/ofae508。
7. Yang ZD, Hu JG, Lu QB, et al. The prospective evaluation of viral loads in patients with severe fever with thrombocytopenia syndrome. J Clin Virol. 2016;78:123-128. doi:10.1016/j.jcv.2016.03.017
8. Ding YP, Liang MF, Ye JB, et al. Prognostic value of clinical and immunological markers in acute phase of SFTS virus infection. Clin Microbiol Infect. 2014;20(11):O870-O878. doi:10.1111/1469-0691.12636
9. Wang D, Cao K, Shen X, Zhang B, Chen M, Yu W. Clinical Characteristics and Immune Status of Patients with Severe Fever with Thrombocytopenia Syndrome. Viral Immunol. Published online June 8, 2022. doi:10.1089/vim.2021.0217
10. Wen Y, Ni Z, Hu Y, et al. Multiple Genotypes and Reassortants of Severe Fever With Thrombocytopenia Syndrome Virus Co-Circulating in Hangzhou in Southeastern China, 2013-2023. J Med Virol. 2024;96(11):e70029. doi:10.1002/jmv.70029
11. Chen K, Sun H, Geng Y, Yang C, Shan C, Chen Y. Ferritin and procalcitonin serve as discriminative inflammatory biomarkers and can predict the prognosis of severe fever with thrombocytopenia syndrome in its early stages. Front Microbiol. 2023;14:1168381. doi:10.3389/fmicb.2023.1168381
12. Wang L, Xu Y, Zhang S, Bibi A, Xu Y, Li T. The AST/ALT Ratio (De Ritis Ratio) Represents an Unfavorable Prognosis in Patients in Early-Stage SFTS: An Observational Cohort Study. Front Cell Infect Microbiol. 2022;12:725642. doi:10.3389/fcimb.2022.725642
13. Henter JI. Hemophagocytic Lymphohistiocytosis. N Engl J Med. 2025;392(6):584-598. doi:10.1056/NEJMra2314005
14. Lu T, Yan H, Luo J, Wang S, Xia Y, Xu X. Elevated thrombosis-related biomarkers as predictors of disease severity and mortality in patients with severe fever with thrombocytopenia syndrome. BMC Infect Dis. 2025;25(1):235. Published 2025 Feb 18. doi:10.1186/s12879-025-10574-6
15. Tang N, Li D, Wang X, Sun Z. Abnormal coagulation parameters are associated with poor prognosis in patients with novel coronavirus pneumonia. J Thromb Haemost. 2020;18(4):844-847. doi:10.1111/jth.14768
16. Asoğlu R, Tibilli H, Afşin A, Türkmen S, Barman HA, Asoğlu E. Procalcitonin is a predictor of disseminated intravascular coagulation in patients with fatal COVID-19. Eur Rev Med Pharmacol Sci. 2020;24(22):11953- 11959. doi:10.26355/eurrev_202011_23856
17. Zhang Z, Hu X, Du Q, et al. Clinical characteristics and outcomes of disseminated intravascular coagulation in patients with severe fever with thrombocytopenia syndrome. BMC Infect Dis. 2025;25(1):508. doi:10.1186/s12879-025-10900-y
18. Hou H, Chen R, Jiang Y, et al. Autoantibody Profiles and Prognostic Significance in Severe Fever With Thrombocytopenia Syndrome (SFTS) Patients. J Med Virol. 2025;97(3):e70266. doi:10.1002/jmv.70266
19. Shirley MH, Sayeed S, Barnes I, Finlayson A, Ali R. Incidence of haematological malignancies by ethnic group in England, 2001-7. Br J Haematol. 2013;163(4):465-477. doi:10.1111/bjh.12562
20. Lee J, Jeong G, Lim JH, et al. Severe Fever with Thrombocytopenia Syndrome Presenting with Hemophagocytic Lymphohistiocytosis. Infect Chemother. 2016;48(4):338-341. doi:10.3947/ic.2016.48.4.338.
21. Shin SY, Cho OH, Bae IG. Bone Marrow Suppression and Hemophagocytic Histiocytes Are Common Findings in Korean Severe Fever with Thrombocytopenia Syndrome Patients. Yonsei Med J. 2016;57(5):1286-1289. doi:10.3349/ymj.2016.57.5.1286.
22. Meng T, Ding W, Lv D, Wang C, Xu Y. Lactate dehydrogenase to albumin ratio (LAR) is a novel predictor of fatal outcome in patients with SFTS: an observational study. Front Public Health. 2024;12:1459712. doi:10.3389/fpubh.2024.1459712
23. Verma N, Chakraverty J, Baweja P, Girotra A, Chatterjee L, Chugh M. Extremely High Ferritinemia Associated with Haemophagocytic Lympho Histiocytosis (HLH). Indian J Clin Biochem. 2017;32(1):117-120. doi:10.1007/s12291-016-0559-8
24. Liu X, Zhang F, Qiao J, He W. Serum IL-6 and IL-10 levels are associated with fatal outcomes in patients with SFTS in China. J Infect Dev Ctries. 2025;19(2):273-279. Published 2025 Feb 28. doi:10.3855/jidc.19939
25. Oh HS, Kim M, Lee JO, et al. Hemophagocytic lymphohistiocytosis associated with SFTS virus infection: A case report with literature review. Medicine (Baltimore). 2016;95(31):e4476. doi:10.1097/MD.0000000000004476
26. Wang G, Ge HH, Hu L, et al. Severe fever with thrombocytopenia syndrome complicated by haemophagocytic lymphohistiocytosis: a retrospective cohort study. Clin Microbiol Infect. 2024;30(4):558-560. doi:10.1016/j.cmi.2024.01.009
27. Lee J, van Es N, Takada T, et al. Covariate-specific ROC curve analysis can accommodate differences between covariate subgroups in the evaluation of diagnostic accuracy. J Clin Epidemiol. 2023;160:14-23. doi:10.1016/j.jclinepi.2023.06.001
28. Hosmer, David W., Stanley Lemeshow, and Rodney X. Sturdivant. Applied Logistic Regression. 3rd ed. Wiley, 2013
29. Caliendo M, Kopeinig S. Some practical guidance for the implementation of propensity score matching. Journal of economic surveys. 2008 Feb;22(1):31-72.
30. Li H, Lu QB, Xing B, et al. Epidemiological and clinical features of laboratory-diagnosed severe fever with thrombocytopenia syndrome in China, 2011-17: a prospective observational study. Lancet Infect Dis. 2018;18(10):1127-1137. doi:10.1016/S1473-3099(18)30293-7
31. Shin J, Kwon D, Youn SK, Park JH. Characteristics and Factors Associated with Death among Patients Hospitalized for Severe Fever with Thrombocytopenia Syndrome, South Korea, 2013. Emerg Infect Dis. 2015;21(10):1704-1710. doi:10.3201/eid2110.141928
32. Zhou J, Zhou J, Shen DT, Goyal H, Wu ZQ, Xu HG. Development and validation of the prognostic value of ferritin in adult patients with Hemophagocytic Lymphohistiocytosis. Orphanet J Rare Dis. 2020;15(1):71. doi:10.1186/s13023-020-1336-6
33. Kim D, Lai CJ, Cha I, Jung JU. Current Progress of Severe Fever with Thrombocytopenia Syndrome Virus (SFTSV) Vaccine Development. Viruses. 2024;16(1):128. doi:10.3390/v16010128
34. Dai ZN, Peng XF, Li JC, et al. Effect of genomic variations in severe fever with thrombocytopenia syndrome virus on the disease lethality. Emerg Microbes Infect. 2022;11(1):1672-1682. doi:10.1080/22221751.2022.2081617
35. Casel MA, Park SJ, Choi YK. Severe fever with thrombocytopenia syndrome virus: emerging novel phlebovirus and their control strategy. Exp Mol Med. 2021;53(5):713-722. doi:10.1038/s12276-021-00610-1
36. Kwon JS, Kim MC, Kim JY, et al. Kinetics of viral load and cytokines in severe fever with thrombocytopenia syndrome. J Clin Virol. 2018;101:57-62. doi:10.1016/j.jcv.2018.01.017；
